# Socio-economic and lifestyle factors associated with overweight in Flemish adult men and women

**DOI:** 10.1186/1471-2458-7-23

**Published:** 2007-02-26

**Authors:** Nathalie Duvigneaud, Katrien Wijndaele, Lynn Matton, Peter Deriemaeker, Renaat Philippaerts, Johan Lefevre, Martine Thomis, William Duquet

**Affiliations:** 1 Department of Human Biometry and Biomechanics, Faculty of Physical Education and Physical Therapy, Vrije Universiteit Brussel, Pleinlaan 2, B-1050 Brussel, Belgium; 2 Department of Movement and Sports Sciences, Ghent University, Watersportlaan 2, B-9000 Gent, Belgium; 3 Department of Biomedical Kinesiology, Faculty of Kinesiology and Rehabilitation Sciences, K.U.Leuven, Tervuursevest 101, B-3001 Leuven, Belgium

## Abstract

**Background:**

Changes in lifestyles and in the environment over the last decades are probably the most important cause of the overweight epidemic, but the findings are inconsistent among studies. The purpose of this study was to investigate the association of several socio-economic and lifestyle factors with overweight in Flemish adults, using BMI ≥ 25 kg/m^2^, waist circumference (WC) ≥ 94 cm (men) or ≥ 80 cm (women) and the combination of BMI and WC for identifying overweight.

**Methods:**

This cross-sectional epidemiological study was conducted by the Flemish Policy Research Centre Sport, Physical Activity and Health between October 2002 and February 2004 in 46 Flemish communities. A total of 4903 Flemish adults (2595 men and 2308 women), aged 18 to 75 years, from a population-based random sample were included in the analysis. Body weight, height and WC were measured, and socio-economic and lifestyle factors were reported by means of validated questionnaires.

**Results:**

The results of the logistic regressions revealed that age is positively associated with overweight in both genders. Alcohol consumption is associated with overweight only in men. Men smoking in the past and watching TV >11 h/week have significantly higher OR's for overweight, while men who participate in health related sports >4 h/week have significantly lower OR's for overweight. In women, watching TV >9 h/week was positively associated with overweight. Women who are current smokers or participate in health related sports >2.5 h/week or with a higher educational level have significantly lower odds for overweight. Different results are observed between the first (BMI) and the second model (WC) in both genders. In men, the models differ for education and health related sports, while in women they differ for smoking status and leisure time physical activity.

**Conclusion:**

The present study confirms the contention that overweight is a multifactorial problem. Age and TV viewing are positively associated with overweight, while educational level and health related sports are negatively related to overweight in both genders. In men, alcohol consumption and smoking in the past are also among the lifestyle factors associated with overweight. This study also indicates that BMI and WC do not have the same discriminative function regarding different lifestyle factors.

## Background

Notwithstanding the growing attention to overweight due to its impact on public health and health care costs, overweight prevalences are still escalating into a global epidemic [1]. The burden of diseases associated with overweight is huge: hypertension, dyslipidemia, insulin resistance, type 2 diabetes, coronary heart disease, ischemic stroke, osteoarthritis and certain types of cancers. Furthermore, the increase of a sedentary lifestyle, associated with the same range of health problems, even worsens the situation in developed countries [1].

To manage the overweight epidemic, it is essential to understand the complex processes leading to the excess of adiposity. These processes involve interactions of numerous factors, including genetic predisposition, social, cultural, environmental, and behavioural factors [2,3]. Although it is commonly accepted that genetic aspects contribute significantly to the variability in body fatness [4], changes in lifestyles and in the environment over the last decades are probably the most important cause of the overweight epidemic [5]. A number of studies have investigated the relationship between socio-demographic and socio-economic factors on the one hand, and overweight on the other hand. Age was found to be significantly associated to overweight [6-9]. In industrialized countries a lower socio-economic status is associated with a higher risk of overweight in women, with a less apparent relationship in men [6,10-12].

Several studies aiming to determine overweight inducing factors, investigated the association of overweight with lifestyle behaviours such as smoking [13-15], alcohol [16-18], dietary habits and physical (in)activity [19-25]. The determinants of overweight are found to be multifactorial and gender specific but the findings are inconsistent among studies. Epidemiological studies, assessing the relationship between alcohol consumption and BMI, revealed contradictory results. Some researchers concluded that alcohol consumption may contribute to overweight [16-18], whereas in another study it was found that moderate alcohol consumption may have a protective effect on overweight [18]. Studies focusing on the association between body weight and smoking generally concluded that body weight seems to be the highest in former smokers, the lowest in current smokers and medium in never smokers [13-15]. A number of studies, but not all, have established that physical activity is inversely associated with body weight, and these results are less consistent for women than for men [19,20,26]. However, there is substantial evidence that the level of physical activity is associated with overweight and it has been suggested that increasing levels of sedentariness, such as TV watching and computer use, have played a major role in the development of the current overweight epidemic [5,21-25]. Sedentary behaviour has been found to increase the risk of overweight [22-24] and type 2 diabetes [22].

Although BMI is an imprecise measurement of fatness [27-29], most studies investigating the association between overweight and the potential related factors, used BMI to define overweight. In a preliminary study (N Duvigneaud et al. – unpublished data), a BMI of 30 kg/m^2 ^was shown to have insufficient sensitivity to screen for excess body fat in Flemish adults. A BMI ≥ 25 kg/m^2 ^and a waist circumference (WC) ≥ 94 cm for men and ≥ 80 cm for women show a better sensitivity. Furthermore, several authors have suggested the combination of BMI and WC as a diagnostic tool for overweight and health risk [30,31].

The main objective of the present study was to investigate the association of several socio-economic and lifestyle factors with overweight in Flemish adults, using BMI ≥ 25 kg/m^2^, WC ≥ 94 cm (men) or ≥ 80 cm (women), and the combination of BMI and WC for identifying overweight.

## Methods

### Survey and subjects

The data for this study were collected by the Flemish Policy Research Centre Sport, Physical Activity and Health (SPAH) between October 2002 and February 2004 in 46 Flemish communities selected by means of a weighted random procedure. One of the main purposes of the SPAH Study was to investigate the actual status and pattern of physical activity, sports participations, physical fitness and general health among the adult population of Flanders (the Northern part of Belgium). This large scale epidemiological study was supported by the Flemish Government.

A total of 4903 Flemish adults (2595 men and 2308 women), aged 18 to 75 years were included in the present study. They were respondents of a larger sample randomly selected within the communities by the National Institute of Statistics. In each community, the size of the random sample was proportionate to its population size. The detailed establishment and description of the sample has been given elsewhere [32]. All subjects signed an informed consent statement before participating in the study. The study was approved by the Ethics Committee of the Ghent University Hospital.

Our sample was compared with the total Flemish adult population to evaluate its representativeness. Although some small differences were observed, our sample can be considered as sufficiently representative for geographic distribution, age, gender and educational level [32].

### Measurements

#### Anthropometry

The anthropometric measurements were taken by trained staff, using standardized procedures and equipment as proposed by the International Society for the Advancement of Kinanthropometry [33]. All measurements were taken with participants wearing minimal clothing. Body height was measured using a stadiometer (Holtain, Crymych, UK) to the nearest 0.1 cm. Body weight was recorded with a digital weighing scale (Seca 841) to the nearest 0.1 kg. Waist circumference (WC) was measured using a metal tape (Rosscraft, Surrey, BC, Canada) to the nearest 0.1 cm, at the narrowest level, between the lowest rib margin and the iliac crest.

#### Assessment of alcohol consumption and smoking status

To asses the lifestyle factors validated computerized questionnaires [34,35] were used. Before completion, participants were instructed on the use of the computerized questionnaires. During completion trained staff members stayed available to answer any possible questions.

The questions concerning alcohol consumption were based on the Belgian National Health Interview Survey, which is a validated instrument used to estimate health related issues in the Belgian population every 5 years [36]. For drinking behaviour, the subjects were categorized as never drinker, moderate drinker (1–3 drinks/day), infrequent heavy drinker (≥ 4 drinks/weekday or weekend day), frequent heavy drinker (≥ 4 drinks every day). The WHO Monica Smoking Questionnaire [37] was used to assess smoking. According to their responses, the participants were classified into 3 groups: those who had never smoked (never smokers), those who had smoked in the past, but had quit smoking (former smokers) and those currently smoking (current smokers).

#### Assessment of educational level and physical activity

The educational level and some physical (in) activity variables were evaluated using the Flemish Physical Activity Computerized Questionnaire (FPACQ). Participants were classified into low (primary school), moderate (secondary school) and high (college or university) education level. The FPACQ was proved to be a reliable and reasonably valid questionnaire for the assessment of different dimensions of physical activity during a usual week in students [38] and in adult employed/unemployed and retired people [39]. According to their responses, 3 variables of physical (in) activity were calculated for this study: time spent in health related sports activities (Tsport), total leisure time physical activity (TLTPA) and time watching TV/using computer (Ttv). Time spent in health related sports activities was assessed by asking respondents to select their 3 most practiced sports activities from a list of 196 sports. For each of these sports activities, frequency (from once/year to more than once/day) and duration (from some h/year to more than 20 h/week) were also reported. For classification of exercise intensity, the MET-value of each sports activity was determined according to Ainsworth et al. [40]. Dependent on age, the sports activities have to meet a certain MET-value to induce health benefits. Therefore, the American College of Sports Medicine (ACSM) recommendations were used to determine the hours of health related sports activities (h/week) [41]. For individuals younger than 35 years, the sport activities should have a MET-value ≥ 4.5. For individuals between 35 and 50 years, a MET-value ≥ 4 is necessary and for individuals of 50 years and older a MET-value ≥ 3.5 is sufficient to induce health benefits. TLTPA (h/week) sums the time spent on all active leisure time activities. This variable includes active transportation (walking and cycling) during leisure time (from 0 to >60 min/day), household and garden activities (from 0 to >42 h/week) and time spent in sports activities. As an indicator of sedentary behaviour, Ttv (h/week) was calculated. Participants were asked to indicate time spent watching television or playing computer or video games during an average weekday and weekend day (from 0 to ≥ 6 h/day).

### Statistics

Data were analysed using the SPSS 13.0 statistical software package for Windows. All analyses were performed by gender. Descriptive statistics were calculated for all variables. Gender effects were tested by t-test or chi-square test. Binary logistic regression models were used to assess the association between overweight and the socio-economic and lifestyle factors. In all models, the dependent variable was overweight. In the first model, the internationally accepted BMI cutoff for overweight (25 kg/m^2^) was used. In the second model, overweight was defined using WC ≥ 94 cm in men and WC ≥ 80 cm in women, as proposed by Lean et al [42]. In the third model, the combination of BMI and WC was used to determine overweight. For the combination of BMI and WC, only subjects with both a BMI ≥ 25 kg/m^2 ^and a WC ≥ 94 cm (men) or ≥ 80 cm (women) were considered as overweight, whereas the subjects with both a BMI <25 kg/m^2 ^and a WC <94 cm (men) or WC <80 cm (women) were considered to have a normal or healthy weight. The subjects with a normal BMI and an increased WC or the inverse were not taken into the analysis. Age was introduced as a continuous variable and all other independent factors of the model were included as categorical variables: alcohol consumption (never, moderate, infrequent heavy, frequent heavy drinker), smoking status (never, former, current), education level (primary, secondary, college or university), Tsport (tertiles), TLTPA (tertiles) and Ttv (tertiles). The results are presented as odd ratios (OR) and 95% confidence intervals (95% CI), adjusted for age and all other variables in the model (alcohol, smoking, education, Tsport, TLTPA, Ttv). Significance level for entry was set at *P *< 0.05 and for removal at *P *< 0.10.

## Results

The descriptive characteristics of the sample are presented in Table 1. Men have significantly higher mean BMI and WC than women. Males participate more in health related sports, work longer and watch more television than females. Flemish females spend more time in leisure time physical activities compared to males.

**Table 1:** Descriptive characteristics (mean ± SD or percentages) in Flemish men and women

	men		women	
	
	N	Mean (SD)	N	Mean (SD)
Age (yrs) **	2595	47.68 (14.77)	2308	46.59 (13.81)
BMI (kg/m^2^) ***	2595	25.82 (3.52)	2308	24.79 (4.15)
WC (cm) ***	2595	90.88 (10.56)	2308	79.06 (10.49)
Health related sports (h/week) ***	2595	3.66 (4.43)	2308	2.40 (3.38)
Total leisure time PA (h/week) ***	2595	18.80 (10.92)	2308	22.90 (10.06)
TV viewing (h/week) ***	2595	15.81 (9.01)	2308	14.89 (8.74)
				
	N	%	N	%

Alcohol consumption ††				
Never	125	4.8	215	9.3
1–3 drinks/day	1738	67.0	1910	82.8
≥4 drinks/week or WEday	596	23.0	169	7.3
≥4 drinks/every day	136	5.2	14	0.6
Smoking status ††				
Never	1326	51.1	1603	69.5
Former	771	29.7	338	14.6
Current	498	19.2	367	15.9
Education NS				
Primary	675	26.0	617	26.7
Secondary	823	31.7	677	29.3
College/university	1097	42.3	1014	43.9
				
	BMI ≥ 25 kg/m^2^	WC ≥ 94 cm	BMI ≥ 25 kg/m^2^	WC ≥ 88 cm

Age category	(%)	(%)	(%)	(%)
18–34 y	30.7	10.6	21.3	17.3
35–54 y	57.2	35.1	37.8	36.9
55–75 y	72.9	58.5	61.1	65.9

Differences between sexes: **P < 0.01 (t-test), ***P < 0.001 (t-test), † *P *< 0.01 (chi-square), ††*P *< 0.001 (chi-square), NS: not significant, WEday: weekend day

About 5% of the males and 9% of the females never drink alcoholic beverages. Most Flemish participants (males: 67%, women: 82.8%) drink moderately. More than a quarter of the men are infrequent or frequent heavy drinkers. In women, this percentage was found to be 7.9%. More male participants are ex-smokers or current smokers compared to females. More than 40% of the subjects of both genders have a college or university degree. The overweight prevalence by age category according to the BMI (≥ 25 kg/m2) and WC (≥ 94 cm for men or ≥ 88 cm for women) cutoffs for overweight are also presented in Table 1. The prevalence of overweight increases with age category and is largely above the 50% in the oldest age category of both genders.

The adjusted OR's for the likelihood of being overweight by socio-economic and lifestyle variables in Flemish men are presented in Table 2. In the first model, BMI ≥ 25 kg/m^2 ^was used to define overweight. In this model, each additional year from 18 to 75 years multiplies the risk of overweight by a factor 1.04. This model also reveals that males drinking 1 to 3 drinks/day (OR = 0.62), males having a college or university degree (OR = 0.69) and males participating in health related sports for more than 4 h/week (OR = 0.79) have significantly lower OR's with regard to overweight. On the other hand, males who stopped smoking (OR = 1.59) and males who spend more than 11 h/week (OR = 1.58) watching TV have significantly higher OR's for being overweight compared to the reference category.

**Table 2:** Odds ratios for the likelihood of being overweight by socio-economic and lifestyle variables in men

	BMI			WC			Combination BMI and WC		
	
	N	OR^1^	(95% CI)	N	OR^1^	(95% CI)	N	OR^1^	(95% CI)
Age	2595	1.04	**p < 0.001**	2595	1.06	**p < 0.001**	2054	1.06	**p < 0.001**
Alcohol consumption			**p = 0.012**			**p = 0.032**			**p = 0.018**
Never	125	1.00	(ref.)	125	1.00	(ref.)	91	1.00	(ref.)
1–3 drinks/day	1738	0.62	(0.41–0.94)*	1738	0.94	(0.62–1.43)	1379	0.71	(0.43–1.16)
≥ 4 drinks/week or WEday	596	0.78	(0.51–1.21)	596	1.19	(0.76–1.86)	474	0.92	(0.55–1.56)
≥ 4 drinks/every day	136	0.94	(0.54–1.63)	136	1.54	(0.89–2.67)	110	1.25	(0.66–2.37)
Smoking status			**p < 0.001**			**p < 0.001**			**p < 0.001**
Never	1326	1.00	(ref.)	1326	1.00	(ref.)	1036	1.00	(ref.)
Past	771	1.59	(1.30–1.96)***	771	1.71	(1.40–2.10)***	615	1.94	(1.54–2.44)***
Current	498	0.88	(0.70–1.11)	498	1.08	(0.84–1.38)	403	0.96	(0.74–1.26)
Education			**p < 0.001**			**p = 0.201**			**p = 0.017**
Primary	675	1.00	(ref.)	675	1.00	(ref.)	533	1.00	(ref.)
Secondary	823	0.97	(0.77–1.23)	823	0.88	(0.70–1.11)	632	0.91	(0.70–1.19)
College or university	1097	0.69	(0.55–0.87)**	1097	0.81	(0.64–1.02)	889	0.71	(0.54–0.92)**
Health related sports (h/week)			**p = 0.080**			**p < 0.001**			**p = 0.009**
Tertile 1 (<0.62)	857	1.00	(ref.)	857	1.00	(ref.)	696	1.00	(ref.)
Tertile 2 (0.62 – 4)	903	0.97	(0.79–1.19)	903	0.75	(0.60–0.93)**	703	0.82	(0.64–1.04)
Tertile 3 (>4)	835	0.79	(0.63–0.99)*	835	0.61	(0.48–0.77)***	655	0.66	(0.50–0.86)**
Total leisurte time PA (h/week)			**p = 0.399**			**P = 0.235**			**p = 0.163**
Tertile 1 (<12.73)	863	1.00	(ref.)	863	1.00	(ref.)	687	1.00	(ref.)
Tertile 2 (12.73 – 20.83)	863	0.87	(0.70–1.07)	863	0.83	(0.66–1.03)	683	0.78	(0.61–1.01)
Tertile 3 (>20.83)	869	0.91	(0.72–1.15)	869	0.86	(0.68–1.10)	684	0.85	(0.64–1.12)
TV viewing (h/week)			**p < 0.001**			**p < 0.001**			**p < 0.001**
Tertile 1 (<11)	820	1.00	(ref.)	820	1.00	(ref.)	657	1.00	(ref.)
Tertile 2 (11 – 19)	931	1.58	(1.29–1.94)***	931	1.45	(1.16–1.81)**	710	1.67	(1.31–2.13)***
Tertile 3 (>19)	844	1.54	(1.24–1.92)***	844	1.80	(1.43–2.27)***	687	1.97	(1.52–2.54)***

^1 ^Odds ratios adjusted for age and all other variables in the table, *P < 0.05, **P < 0.01, *** *P *< 0.001, WEday: weekend day

In the second model, using WC ≥ 94 cm to determine overweight, educational level (p = 0.201) is not significantly associated with overweight. Alcohol consumption as a global factor is significant (p = 0.032), but none of the alcohol consumption levels reaches significance. Each additional year of age multiplies the risk of having WC ≥ 94 cm by 1.06. Similar to the first model, males who smoked in the past have 71% higher odds of being overweight than males who never smoked, and males watching TV more than 11 h/week also have significantly higher OR's compared to the reference category. Males in the second and third tertile of health related sports have significantly less chance of being overweight compared to males in the reference category, OR's 0.75 and 0.61 respectively.

In the third model, overweight was defined by the combination of BMI ≥ 25 kg/m^2 ^and WC ≥ 94 cm. As in the second model, for each additional year the risk of overweight is multiplied by a factor 1.06, while the levels of alcohol consumption are not significantly associated with the likelihood of being overweight. According to this last model, former male smokers (OR = 1.94) and males watching TV more than 11 h/week (OR = 1.67) and more than 19 h/week (OR = 1.97) have also significantly higher OR's for being overweight compared to the reference category. In all 3 models, TLTPA is not significantly associated with overweight.

The adjusted OR's for the likelihood of being overweight by socio-economic and lifestyle variables in Flemish women are given in Table 3. In all three models, age is positively associated with the risk of being overweight. Each additional year of age from 18 to 75 years multiplies the risk of being overweight by 1.04 in the first model or by 1.06 in the second and third model. Alcohol consumption (p = 0.701) is not significantly associated with overweight in women. In the first model, females who are currently smoking (OR = 0.66), females having a secondary (OR = 0.74) or college/university degree (OR = 0.56) and females participating in health related sports more than 2.46 h/week (OR = 0.71) have significantly lower OR's for being overweight (BMI ≥ 25 kg/m^2^) compared to the reference category. Females in the third tertile of TLTPA and females who spend more than 9 h/week watching TV have 29% higher odds of being overweight compared to the reference category.

**Table 3:** Odds ratios for the likelihood of being overweight by socio-economic and lifestyle variables in women

	BMI			WC			Combination BMI and WC		
	
	N	OR^1^	(95% CI)	N	OR^1^	(95% CI)	N	OR^1^	(95% CI)
Age	2308	1.04	**P < 0.001**	2308	1.06	**p < 0.001**	2019	1.06	**p < 0.001**
Alcohol consumption			**P = 0.701**			**p = 0.177**			**p = 0.289**
Never	215	1.00	(ref.)	215	1.00	(ref.)	190	1.00	(ref.)
1–3 drinks/day	1910	0.91	(0.67–1.24)	1910	0.84	(0.61–1.15)	1664	0.87	(0.62–1.22)
≥ 4 drinks/week or WEday	169	0.98	(0.62–1.55)	169	0.94	(0.59–1.50)	153	0.97	(0.59–1.60)
≥ 4 drinks/every day	14	1.59	(0.50–5.07)	14	2.63	(0.78–8.92)	12	2.65	(0.73–9.63)
Smoking status			**P = 0.005**			**p = 0.053**			**p = 0.013**
Never	1603	1.00	(ref.)	1603	1.00	(ref.)	1394	1.00	(ref.)
Past	338	1.06	(0.83–1.36)	338	1.15	(0.89–1.48)	295	1.14	(0.87–1.51)
Current	367	0.66	(0.51–0.86)**	367	0.77	(0.59–1.00)	330	0.69	(0.52–0.92)*
Education			**P < 0.001**			**p = 0.001**			**p < 0.001**
Primary	617	1.00	(ref.)	617	1.00	(ref.)	531	1.00	(ref.)
Secondary	677	0.74	(0.59–0.94)*	677	0.74	(0.58–0.94)*	582	0.70	(0.54–0.91)**
College or university	1014	0.56	(0.44–0.71)***	1014	0.62	(0.48–0.80)***	906	0.55	(0.42–0.72)***
Health related sports (h/week)			**P = 0.013**			**p = 0.005**			**p = 0.002**
Tertile 1 (0)	772	1.00	(ref.)	772	1.00	(ref.)	687	1.00	(ref.)
Tertile 2 (0 – 2.46)	776	0.87	(0.70–1.08)	776	0.92	(0.71–4.15)	686	0.86	(0.68–1.10)
Tertile 3 (>2.46)	760	0.71	(0.57–0.89)**	760	0.69	(0.54–0.87)**	646	0.63	(0.49–0.82)***
Total leisurte time PA (h/week)			**p = 0.114**			**p = 0.045**			**p = 0.041**
Tertile 1 (<22.49)	765	1.00	(ref.)	765	1.00	(ref.)	692	1.00	(ref.)
Tertile 2 (22.49 – 39.56)	772	1.10	(0.88–1.38)	772	0.91	(0.72–1.14)	672	0.97	(0.76–1.25)
Tertile 3 (>39.56)	771	1.29	(1.01–1.64)*	771	1.21	(0.94–1.54)	655	1.31	(1.00–1.72)*
TV viewing (h/week)			**p = 0.079**			**p = 0.017**			**p = 0.010**
Tertile 1 (<9)	562	1.00	(ref.)	562	1.00	(ref.)	506	1.00	(ref.)
Tertile 2 (9 – 18)	1014	1.29	(1.02–1.62)*	1014	1.41	(1.11–1.79)**	892	1.41	(1.09–1.82)*
Tertile 3 (>18)	732	1.29	(0.99–1.67)*	732	1.35	(1.04–1.77)*	621	1.39	(1.04–1.86)*

^1 ^Odds ratios adjusted for age and all other variables in the table, *P < 0.05, **P < 0.01, *** *P *< 0.001, WEday: weekend day

In the second model, alcohol consumption (p = 0.177), smoking status (p = 0.053) and levels of TLTPA are not significantly associated with overweight defined by WC ≥ 80 cm. Similar to the first model, females with a secondary (OR = 0.74) or college/university degree (OR = 0.62) and females participating in health related sports activities more than 2.46 h/week (OR = 0.69) have less chance of being overweight compared to the reference category. Females in the second and third tertiles of watching TV have significantly higher OR's, 1.41 and 1.35 respectively, for being overweight compared to the females in the first tertile.

The third model, using the combination of BMI ≥ 25 kg/m^2 ^and WC ≥ 80 cm shows results similar to model 1 in women.

## Discussion

The main purpose of the present study was to determine the association of several socio-economic and lifestyle factors with overweight in Flemish adults, using BMI ≥ 25 kg/m^2^, WC ≥ 94 cm (men) or WC ≥ 80 cm (women) and the combination of BMI and WC for identifying individual overweight.

Although BMI has some limitations, most studies investigating the overweight associated factors used this index as sole indicator of overweight. One of the strengths of this study is the added use of WC, next to BMI, as an indicator of abdominal obesity. Although BMI shows a high positive correlation with WC in men (r = 0.91) and women (r = 0.90), different results were observed between the first (BMI) and the second model (WC) in both genders. In men, the models differ for education and health related sports, while in women they differ for smoking status and TLTPA. This finding indicates that BMI and WC have not the same discriminative function regarding the different lifestyle factors. The OR's based on the combined use of BMI and WC are somewhat more explicit. This may be due to the fact that the combined use of BMI and WC allows to distinguish between the overweight and non-overweight group more accurately because doubtful cases were excluded. All excluded men (20.8%) have a normal WC, but a BMI ≥ 25 kg/m2. Only 12.5% of the women were excluded, half of them for an increased BMI and a normal WC, the other half for a normal BMI, but an increased WC.

In agreement with the literature, age was positively related with overweight in both genders. The results of epidemiological studies on the association between alcohol intake and body weight are equivocal. A recent study of Breslow and Smothers [17], examining the association between drinking patterns and BMI, revealed a positive association. Men and women who consumed the smallest quantity of alcohol per drinking day had the lowest BMI, those who consumed the greatest quantity had the highest BMI. The expectation that consumption of alcohol might be associated with overweight was not fully confirmed in the present study. Only frequent heavy drinkers (≥ 4 drinks/every day) are more likely to be overweight in both genders. The small number of frequent heavy drinkers in female subjects may have contributed to insufficient statistical power to detect significance. On the other hand, males consuming 1 to 3 drinks per day have a significantly lower OR (0.62) for overweight compared to never drinkers in the first model. Similar to our findings, other studies have reported that moderate drinking appears not to be positively associated with overweight in both genders [18,19,43,44]. An explanation for the U-shape relationship between alcohol intake and overweight may be that moderate drinkers of alcoholic beverages compensate for energy derived from alcohol by eating less [17].

Smoking is usually associated with lower BMI. According to several authors, body weight appears to be the highest in ex-smokers, and the lowest in current and medium in never smokers [13-15,45]. Our results corroborate these findings. Using BMI and the combination method to define overweight, women who are current smokers have significantly lower odds for being overweight. However, this trend was not significant in men. Former smokers in Flemish men had significantly higher OR for overweight compared to never and current smokers in all 3 models. The same but not significant trend was observed in women. It is suggested that the weight gain associated with smoking cessation could be partly caused by the lack of nicotine as an appetite suppressant [11]. Smoking cessation also leads to changes in adipose cell metabolism, in particular increases in adipose tissue lipoprotein lipase activity [46,47]. This process may also contribute to the increase in weight gain associated with smoking cessation. Given the well-known smoking health related risks, but also given the expected weight gain associated with smoking cessation, anti-smoking campaigns should especially target youth to prevent them to start smoking.

Overall, the prevalence of overweight and obesity in developed countries is higher in lower socio-economic groups [9,48-50]. In the present study, women with higher level of education (secondary or college/university diploma) are less likely to be overweight in all three models. As in other studies [51-53], the relationship between education level and overweight was less consistent in men. It has been suggested that individuals with higher education tend to have healthier behaviours, including healthier dietary habits than those with low education [15,53,54]. A higher educational level may act upon overweight through better knowledge of healthy food habits or through more comfortable budgetary conditions to buy healthy nutrients such as fruits and vegetables.

An explanation for the fact that females in the highest tertile of TLTPA are more likely to be overweight according to the first and the third model, may be that TLTPA also includes activities of low intensity (e.g. housekeeping) not affecting weight and body composition. On the other hand, TLTPA is not significantly associated with the likelihood of being overweight among Flemish men. Similarly, in the study of Santos et al. [26] no significant contribution of total physical activity was found when comparing obese to normal weight participants. However, when only regular physical exercise was considered, obese participants of both genders were found to take significantly less exercise. Several authors have reported an inverse association of self-reported physical activity with obesity and increasing BMI [19,20,26,53]. Similar results are also observed in our study. Adults in the highest tertile of health related sports have significantly lower OR's for the likelihood of being overweight in all three models.

As in numerous other studies [5,21-25], our results indicate a positive association between watching TV/using computer and overweight. Flemish males watching TV/using computer more than 11 h/week and females watching TV/using computer more than 9 h/week have significantly higher odds for being overweight. Watching television could lead to overweight through reduced energy expenditure or through the association of television viewing with the consumption of snacks [22]. Due to the limitations in mobility and the social isolation associated with overweight, it is also possible that overweight may lead to more TV viewing.

The present study has some limitations. The cross-sectional design of this study does not permit to infer causal relationships from our results. In addition, the use of questionnaires to assess habitual physical activity has been reported to be crude and imprecise, since it is a less objective measurement of physical activity than accelerometers [55] or pedometers [56,57]. Notwithstanding this critique, the FPACQ used in our study was found to be a reliable and reasonably valid questionnaire for the assessment of different dimensions of physical activity in students [38] and adults [39]. Considering the fact that the development of obesity is mainly due to an imbalance between food intake and energy expenditure, the lack of data concerning dietary habits can also be considered as a limitation of this study.

In spite of its limitations, the present study provides unique data on the socio-economic and lifestyle factors associated with overweight in Flemish adults. Moreover, the data are from a large sample with a wide age range from 18 to 75 years. Another strength of this study is that body weight, height and WC were measured by trained staff and not self-reported. In addition, the combination of BMI and WC, aiming to reduce misclassification of individuals, is an interesting and novel approach to study associations between overweight and lifestyle factors. Combining BMI and WC can be seen as a statistical limitation because the continuity of a one-factor criterion is lost. However, it can also be taken as a methodological improvement as it leads to more contrasting groups. Some associations of lifestyle factors with overweight, found with the combination method, were not detected when using only one single criterion for overweight.

## Conclusion

In conclusion, the present study supports the contention that overweight is a multifactorial problem. The results show that smoking status, education level, time spent in health related sports activities and sedentary behaviour (watching TV/using computer) are associated to the likelihood of being overweight. There is also some evidence that the underlying socio-economic and lifestyle factors associated with overweight may differ between men and women. Our findings also indicate that BMI and WC have not always the same discriminative function for the different lifestyle factors. Finally, the results of the present study support the combined use of BMI and WC to determine overweight and to investigate the socio-economic and behavioural factors associated with overweight.

## Competing interests

The author(s) declare that they have no competing interests.

## Authors' contributions

ND, LM, KW and PD participated in the data collection. WD, JL, RP and MT helped with the study design. ND analysed the data and wrote a first version of the manuscript. All authors provided comments on the drafts and assisted in editing the manuscript. They all read and approved the final version of the manuscript.

## Pre-publication history

The pre-publication history for this paper can be accessed here:


